# Optical nonlinearity enhancement with graphene-decorated silicon waveguides

**DOI:** 10.1038/srep45520

**Published:** 2017-04-12

**Authors:** Atsushi Ishizawa, Rai Kou, Takahiro Goto, Tai Tsuchizawa, Nobuyuki Matsuda, Kenichi Hitachi, Tadashi Nishikawa, Koji Yamada, Tetsuomi Sogawa, Hideki Gotoh

**Affiliations:** 1 NTT Basic Research Laboratories, NTT Corporation, 3-1 Morinosato Wakamiya, Atsugi-shi, Kanagawa, 243-0198, Japan; 2 NTT Nanophotonics Center, NTT Corporation, 3-1 Morinosato Wakamiya, Atsugi-shi, Kanagawa, 243-0198, Japan; 3 NTT Device Technology Laboratories, NTT Corporation, 3-1 Morinosato Wakamiya, Atsugi-shi, Kanagawa, 243-0198, Japan; 4 Tokyo Denki University, Department of Electrical and Electronic Engineering, 5 Senjyu Asahi-cho, Adachi-ku, Tokyo, 120-8551, Japan

## Abstract

Broadband on-chip optical frequency combs (OFCs) are important for expanding the functionality of photonic integrated circuits. Here, we demonstrate a huge local optical nonlinearity enhancement using graphene. A waveguide is decorated with graphene by precisely manipulating graphene’s area and position. Our approach simultaneously achieves both an extremely efficient supercontinuum and ultra-short pulse generation. With our graphene-decorated silicon waveguide (G-SWG), we have achieved enhanced spectral broadening of femtosecond pump pulses, along with an eightfold increase in the output optical intensity at a wavelength approximately 200 nm shorter than that of the pump pulses. We also found that this huge nonlinearity works as a compressor that effectively compresses pulse width from 80 to 15.7 fs. Our results clearly show the potential for our G-SWG to greatly boost the speed and capacity of future communications with lower power consumption, and our method will further decrease the required pump laser power because it can be applied to decorate various kinds of waveguides with various two-dimensional materials.

On-chip coherent ultra-broadband light sources, known as broadband on-chip OFCs, are recognized as indispensable tools in the application fields of chemical-biological spectroscopy^1^,^2^, broadband molecular sensing^3^, waveform generation^4^, and high-capacity optical communications^5^,^6^. In order to generate broadband on-chip OFCs, nonlinear phenomena such as self-phase modulation (SPM), cross-phase modulation, four-wave mixing, self-steepening, stimulated Raman scattering (SRS), Cherenkov radiation, and soliton fissions have to be considered. Over the past few decades, optical fibres featuring various materials and geometries have been investigated for improving the nonlinear coefficient *n*_*2*_, effective mode area size *A*_*eff*_, and dispersion distribution. The synergetic effect of highly nonlinear materials and photonic crystal structures (e.g., germanium-doped silica cores^7^,^8^,^9^, zirconium-fluoride-based fibres^10^,^11^, and chalcogenide glasses fibres^12^) have maximized the nonlinear performance, which has led to spectra broadening ranging from deep UV to mid-IR. Recently, CMOS-compatible broadband light sources based on silicon (Si) and silicon nitride (Si_3_N_4_) have emerged because these materials can be monolithically integrated with electrical circuits by using the nanometers-scale process rule. Indeed, the remarkable advantages are not limited to functionality or scalability, but also provide excellent properties such as a high refractive index and high third-order nonlinearity (*n*_*2,Si*_ of ~10^−18^ m^2^/W^13^,^14^; *n*_*2,Si3N4*_ of ~10^−19^ m^2^/W^15^). Much effort has been focused on improving the efficiency of the broadband on-chip OFC generation from a waveguide by controlling the intrinsic parameters of the waveguide itself, such as its materials, size, and structure^4^,^16^.

Here, we focused on graphene, a two-dimensional material, for obtaining a huge enhancement of the nonlinear optical properties. The waveguide is decorated with graphene by precisely manipulating the graphene’s area and position. In this study, we achieved locally enhanced on-chip OFC generation with the G-SWG. The local Kerr coefficient n_2_(r) is 4.2 × 10^−18^ m^2^/W in bulk silicon^17^ and ~10^−13^ m^2^/W for graphene^18^. As a result, the effective 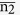 of G-SWG is calculated to be 4.1 × 10^−17^ m^2^/W, which is one order of magnitude higher than that of a SWG. Moreover, both the flat material dispersion in the complex refractive index from the visible to terahertz range and ultra-fast response due to the unique gapless Dirac cone^19^ contribute to the on-chip OFCs’ generation span. In the physical interpretation, graphene itself has extremely large nonlinear optical effects induced by electron interactions and transitions. Recent theoretical analyses of frequency dependent third-order conductivity^20^,^21^,^22^ have revealed that the maximum third-order nonlinearity is expected to be found at the Dirac point, or low-doping regime, due to the dominant contribution of the interband transitions. The intraband transitions play a role in determining the magnitude of Kerr coefficient *n*_2_ as the Fermi level increases in the high-doping regime. The SPM effect with a hybrid structure of graphene on a silicon rib-waveguide has recently been characterized^23^. Though the approach has some similarities to ours, it neglects dispersion engineering, which is the most critical factor for broadband on-chip OFC generation. As a result, only a drastic deformation or splitting of the incident femtosecond pulse is observed instead of a seamless spectrum broadening effect. We propose a different approach: controlling the graphene interaction length and position, the geometry of the waveguide structure, and dispersion with sophisticated techniques developed for the low-dimensional materials integration.

## Results

### Experimental set-up for broadband on-chip OFC generation

**Figure 1:**
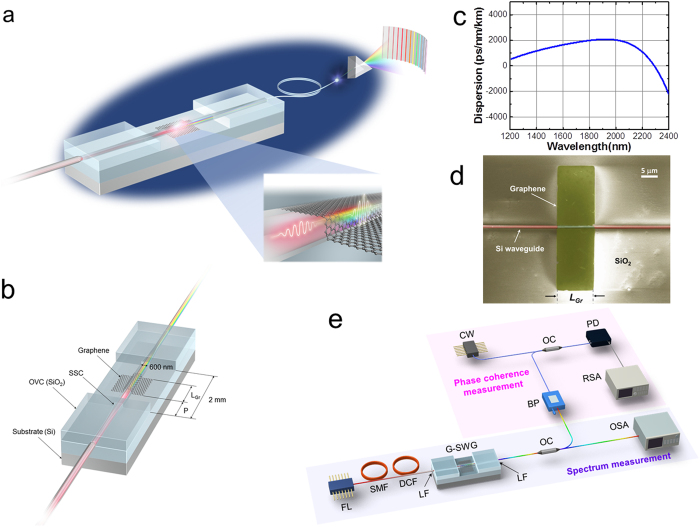
(**a**) A graphene-decorated silicon waveguide (G-SWG) shows a huge local nonlinearity enhancement. (**b**) Bird’s-eye view of the incident side of G-SWG. L_Gr_: graphene length. P: the graphene’s position from the SWG incident side. OVC: over cladding. SSC: spot-size converter. Total length of the SWG is 2 mm. (**c**) Dependence of G-SWG dispersion on wavelength. (**d**) Scanning electron microscope image of G-SWG. (**e**) Experimental setup. The purple and pink regions show the arrangements for the spectrum and phase coherence measurement, respectively. FL: femtosecond mode-locked Er-fibre laser. SMF: single-mode fibre. DCF: dispersion-controlled fibre. LF: lensed fibre. OC: optical coupler. BP: bandpass filter. CW: continuous-wave laser. OSA: optical spectral analyzer. PD: photodetector. RSA: RF spectral analyzer.

Figure 1a illustrates the huge local nonlinearity enhancement in G-SWGs and broadband on-chip OFC generation. The graphene on the SWG plays two important roles—it increases the effective 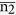 and compresses laser pulse width. Broadband on-chip OFCs can be generated efficiently and can expand the functionality of photonic integrated circuits. A geometric schematic of the designed sample is shown in Fig. 1b. A femtosecond pump pulse light source at 1560 nm is focused on the edge of a 400-μm-long spot-size converter (SSC). This partially window-open SSC prevents film stress and damage to the graphene. It also achieves very efficient light-source coupling between an optical fibre and graphene photonic device on a silicon photonics platform^24^. By optimizing the waveguide structure (tip width: 200 nm), the optical coupling loss is only 1.4 dB/facet for the TE mode. The light propagates through a 600-nm-wide and 220-nm-thick SWG, which works as an introductory region with an initial propagation length (*P*) of 100 to 300 μm. Since the SWG itself also has strong dispersion and negligible third-order nonlinearity, this entrance margin contributes as a regulator for controlling the chirping of incident light pulse. Note that the total device length is fixed at 2 mm. Once the ultra-short pulse reaches the G-SWG in the center of the device, a gigantic nonlinear effect is induced on the SWG, which can be observed as spectrum broadening in the frequency domain through the output side the SSC. Figure 1c shows G-SWG dispersion. The dispersion was calculated with a mode solver (Mode Solution, Lumerical Solutions, Inc.) and was controlled to be anomalous, which can efficiently generate a broadband on-chip OFC spectrum at the pump laser’s wavelength^26^. In addition, the anomalous dispersion can counterbalance the SPM-induced dispersion of the laser pulse in G-SWGs. The dispersive complex refractive index of graphene^27^ was used to calculate the dispersion curves for the hybridized structure of G-SWGs in the TE-like mode. Using these calculation results, we fabricated a 600-nm-wide G-SWG, in which a 10 μm (*L_Gr_*) × 30 μm graphene pattern was defined by O_2_-plasma etching (see Fig. 1d). The calculated SWG width dependence of dispersion and group velocity showed that the group velocity becomes flat over a wide wavelength range when the width is set from 600 to 700 nm in the TE mode (m = 0). However, the weaker interaction between the evanescent field and graphene and the dispersion change in the 700-nm wide SWG can excite an extra mode (m = 1), which must be avoided because of the weaker interaction between the evanescent field and graphene and the dispersion change. This optimization process was done initially by simulation. Therefore, we fabricated the G-SWG at an optimized width of 600 nm and succeeded in generating broadband on-chip OFC efficiently. The other parameters of 100 and 200 μm as the interaction length *L_Gr_* were also designed to investigate the tradeoff between the impact of graphene’s strong nonlinearity and pump pulse absorption or deformation. By optimizing the thermal history control during the decoration process, the graphene on the SWG almost completely covers the sidewalls on both sides, which was confirmed from a high-resolution SEM image (see Supplement Information for more details about the fabrication process and for μ-Raman spectroscopy results). Figure 1e shows the setup for generating the broadband on-chip OFC spectrum in the G-SWG and measuring both the spectrum and phase coherence. The G-SWG is pumped by a femtosecond mode-locked Er-fibre laser oscillator (Menlo Systems GmbH). The laser produces light pulses with a center wavelength of 1560 nm at a repetition rate of 250 MHz, which are delivered with a polarization-maintaining fibre. By adjusting the dispersion with the dispersion-controlled fibre, the pulse width can be compressed to 80 fs. We measured the 80-fs pulse width using a frequency-resolved optical gating. The femtosecond laser pulse is coupled into the G-SWG by using a high numerical aperture (NA = 0.29) lensed fibre with a focal length of 20 μm and anti-reflection coating. The polarization is set to the TE (horizontal) mode of the G-SWG with a rotary fibre holder. The input pulse energy is 120 pJ. For efficient coupling between the G-SWG and Er-doped fibre laser, we defined the SSCs with a partial air-cladding window^24^ at the center of the G-SWG for protection against physical or chemical damage to the graphene sheet during the fabrication processes. The coupling loss at the input waveguide facet and the coupled pulse energy are estimated to be 1.5 dB and 84 pJ, respectively. The measured propagation loss of the G-SWG is 0.052 dB/μm. The output from the waveguide is collected with the lensed fibre and delivered to an optical spectrum analyzer.

### Spectral broadening in the graphene-length-controlled G-SWG

**Figure 2:**
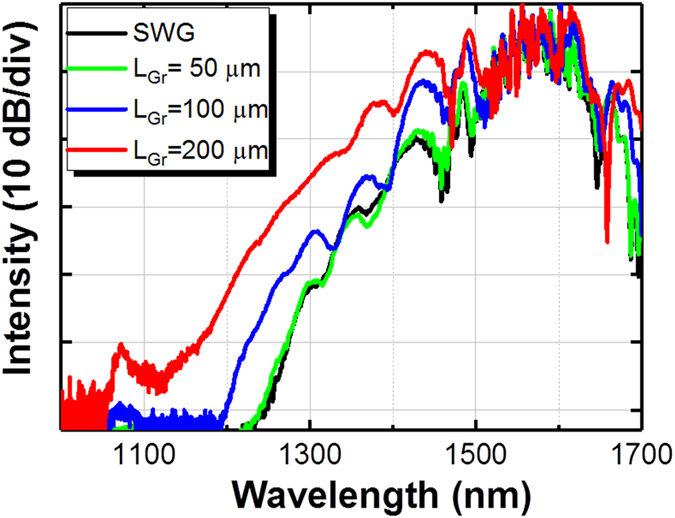
Dependence of the on-chip OFC spectrum on 600-nm-wide G-SWGs (graphene length L_Gr_: 50-, 100-, and 200-μm) with an inverse taper. The black line shows the on-chip OFC spectrum for the SWG without graphene.

Precise manipulation of the graphene length on the SWG plays an important role in achieving the large nonlinear phase shift. We experimentally investigated the characteristics of the broadband on-chip OFC spectra with and without the graphene on the SWG. Figure 2 shows the dependence of the broadband on-chip OFC spectrum on graphene length *L_Gr_* (see Fig. 1b for the definition of *L_Gr_*). The measurement range in our spectral analyzer is limited to less than 1700 nm. The on-chip OFC intensities of each graphene length in Fig. 2 compensate for the propagation loss with the graphene on the SWG. When the graphene length L_Gr_ is less than 50 μm, the on-chip OFC bandwidth is little different from that without the graphene. However, the broadband on-chip OFC spectrum is generated when the graphene length L_Gr_ is more than 100 μm. We found that with current experimental setup, the widest on-chip OFC spectrum (1050–1700 nm) at −60 dB level is generated when the graphene length *L_Gr_* is 200 μm and graphene position *P* is 300 μm from the SWG incident side. Since the band gap of 1.1 eV in silicon translates into an absorption edge of 1100 nm, the on-chip OFC spectrum spans up to the absorption edge. The wavelength dependent loss spectrum can be found in our previous paper^28^, which explained how much linear absorption is induced by graphene in the quasi-TE mode. A wavelength span of 50 nm (1525–1575 nm) had about 2-dB loss increase at the long wavelength (note that this data was characterized by a 400-nm-wide waveguide only). Therefore, the observed OFC spectra in Fig. 2 might include this trend. Ultra-broad-bandwidth coupling from O- to U-bands (1300–1650 nm) has been achieved within only ~2 dB insertion loss^24^. We expect that this wavelength dependence induced by the SSC would be negligibly small.

**Figure 3:**
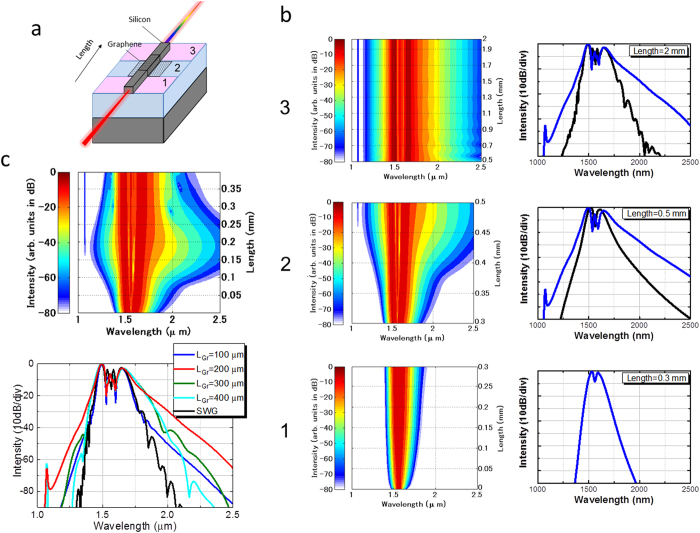
(**a**) G-SWG divided into three areas, with graphene transferred only onto Area 2 of the SWG. (**b**) Left: Simulated spectrum evolution in G-SWG (left). Right: Simulated on-chip OFC spectra with (blue) and without (black) graphene on the SWG for propagation lengths of 0.3, 0.5, and 2 mm (right). (**c**) Left: Simulated spectral evolution in Area 2 of the G-SWG. Right: On-chip OFC spectrum in Area 2 of the G-SWG for the graphene length L_Gr_ of 100 (blue), 200 (red), 300 (green), and 400 μm (light blue) and without graphene on the SWG (black).

By using the nonlinear Schrödinger equation with the split-step Fourier method^29^ (see Methods), the on-chip OFC generation can be simulated. We use a simple model that does not contain SRS and the free carrier effect (FCA) because both the SRS and FCA play minor roles during the on-chip OFC formation in silicon waveguides^30^,^31^. The Raman-gain spectrum of silicon is narrow and the femtosecond laser pulse propagates before free carriers accumulate. We calculated the case where a Gaussian-shape laser pulse is coupled into the G-SWG without an inverse taper. We assumed that the coupled pulse energy becomes lower (40 pJ) than the value estimated from the experiment and the pulse width becomes broader (120 fs) than the input pulse width used in the experiment because of the influence of coupling loss, two-photon absorption (TPA), and frequency chirp at an inverse taper. We divide the G-SWG into three areas as shown in Fig. 3a. Area 1 and 3 are SWG. The graphene is transferred only onto Area 2 of the SWG. Figure 3b and c show the simulated spectral evolution in the G-SWG. When the propagation length of the laser pulse is less than 50 μm in Area 2, the spectrum does not broaden. The spectrum gradually becomes wider as the propagation length exceeds 50 μm. When the propagation length of the laser pulse is 200 μm in Area 2, the on-chip OFC spectrum bandwidth becomes widest and is much wider than in the SWG without graphene. These simulation results are consistent with the experimental results and indicate that the process of broadband on-chip OFC light generation in the G-SWG with a femtosecond laser in the 1.5-μm band can be explained well enough by three nonlinear optical effects—SPM, self-steepening, and TPA.

**Figure 4:**
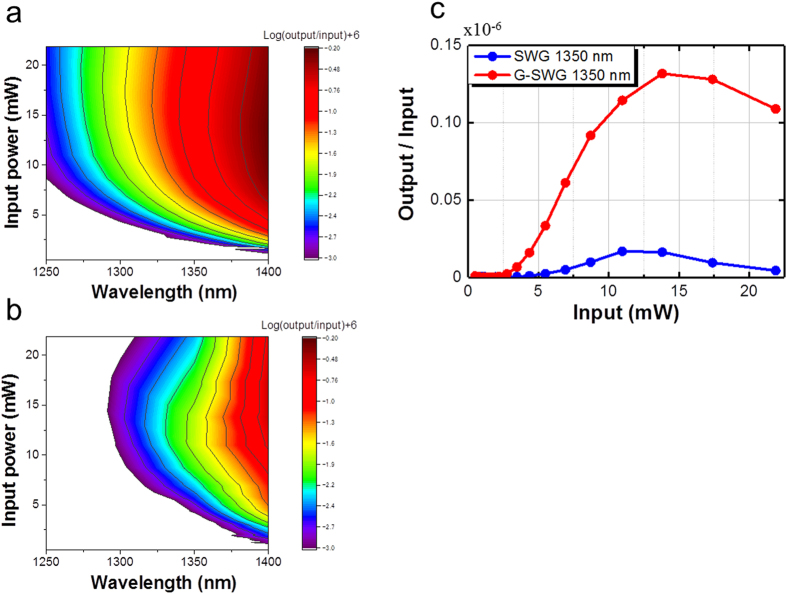
Dependence of the on-chip OFC spectrum on the input laser power (**a**) with and (**b**) without the 200-μm-long graphene on the SWG (graphene length L_Gr_: 200 μm) in the experiment, and (**c**) at the wavelength of 1350 nm in the experiment.

Furthermore, we experimentally investigated the input laser power dependence of the on-chip OFC intensity. Figure 4 shows the generated SC power at each wavelength component divided by the input seed laser power of 1560 nm. It is clearly seen that for every wavelength component, the slopes with the G-SWG become steeper than those without the graphene on the SWG. These results indicate that the G-SWG has a much higher effective 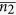 and becomes more sensitive for the input laser power. The negative slope with the input laser power of more than 15 mW might show the influence of TPA.

### Spectral broadening in the graphene-position-controlled G-SWG

**Figure 5:**
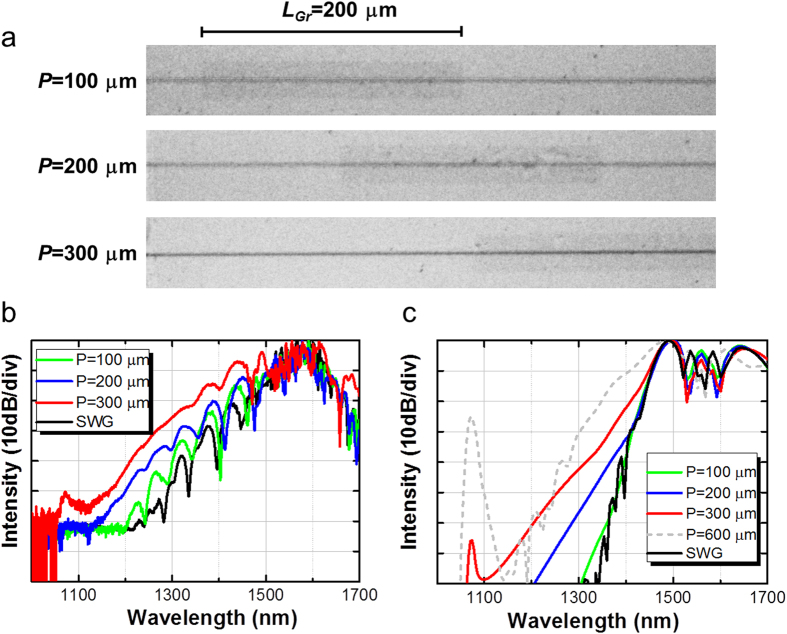
(**a**) Optical images of G-SWGs for the graphene position *P* from 100 to 300 μm in case of length *L_Gr_* 200 μm. Dependence of the on-chip OFC spectrum on graphene 100, 200, and 300 μm from the SWG incident side in (**b**) experiment and (**c**) simulation. The black line shows the on-chip OFC spectrum without the graphene on the SWG. The gray dashed line in the simulation shows the on-chip OFC spectrum for the graphene position of 600 μm.

**Figure 6:**
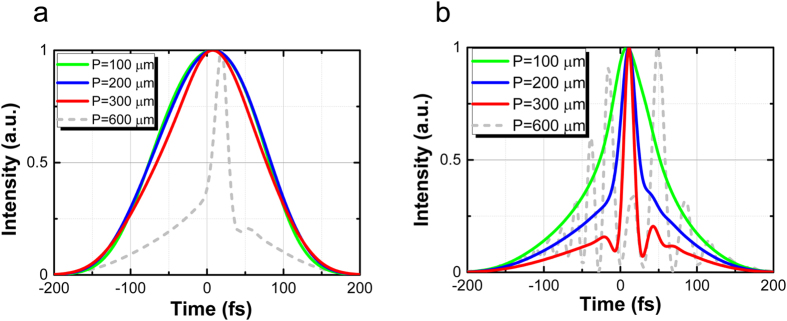
Simulated laser pulse width (**a**) before and (**b**) after Area 2 for graphene position *P* of 100, 200, 300, and 600 μm.

Precise manipulation of the graphene position on the SWG plays an important role in generating short laser pulses with control of the initial chirping. We experimentally investigated the graphene position on the SWG at which the anomalous dispersion in G-SWG can counterbalance SPM-induced dispersion of the laser pulse. Figure 5 shows the dependence of the broadband on-chip OFC spectrum on the graphene position on the SWGs for input pulse energy of 120 pJ. We manipulate the graphene positioned 100, 200, and 300 μm from the SWG incident side in case of length *L_Gr_* of 200 μm as shown in Fig. 5a. The on-chip OFC intensities at each graphene position in Fig. 5b compensate for the propagation loss with the graphene on the SWG. We found that the largest on-chip OFC enhancement can be measured when the graphene is positioned 300 μm from the SWG incident side. We then simulated the dependence of the on-chip OFC intensity on the graphene position using the generalized nonlinear Schrödinger equation with the split-step Fourier method^29^ (see Methods) as shown in Fig. 5c. The simulation results agree quite well with the experimental results. In addition, we calculated the laser pulse shapes in the waveguides when we changed graphene position *P* from 100 to 600 μm. The difference in laser pulse widths in front of the graphene on the SWG is small, while the difference in the phases is large (see Supplementary Information). Figure 6 shows simulated laser pulse width before and after Area 2 (see Fig. 3a) when graphene position *P* was changed from 100 to 600 μm. The laser pulse width becomes shorter as the laser propagates into the graphene-decorated area because the anomalous dispersion of the SWG can counterbalance the SPM-induced dispersion of the laser pulse. Notice that the graphene on the SWG plays an important role in generating the ultra-short pulse—without the graphene, such an ultra-short pulse cannot be generated. We found that the laser pulse width becomes smallest in our prepared G-SWGs when graphene position *P* is 300 μm. The laser pulse width at *P* of 300 μm can be compressed from 143.6 to 15.7 fs before and after Area 2. In Area 2, the effective 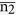 is very large and has a broadband anomalous dispersion region. Therefore, as a result of the SPM, the short laser pulse leads to the spectral broadening. Although the on-chip OFC spectrum becomes wider at *P* of 600 μm, the laser pulse is split. We found from the simulation that the widest and largest on-chip OFC spectrum without splitting the laser pulse can be generated at *P* of 300 μm from SWG incident side (see Supplementary Information).

### Phase-coherent supercontinuum

**Figure 7:**
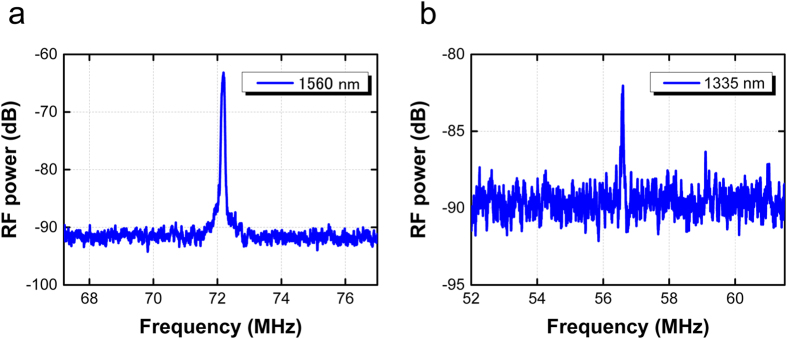
RF spectrum of the beat signals between the on-chip OFC and the tunable laser at (**a**) 1560 and (**b**) 1335 nm.

We experimentally investigated the phase coherence of the on-chip OFC light generated in the G-SWG using beat note measurements with narrow linewidth continuous-wave (CW) lasers as shown in Fig. 1e. The beat note was detected with a balanced photodetector and monitored on an RF spectrum analyzer. Figure 7 shows the beat note of the on-chip OFC light and (a) a free-running CW laser (Koshin Kogaku, linewidth 16 kHz) at 1560 nm and (b) a free-running CW laser (Hewlett Packard, linewidth 100 kHz) at 1335 nm, respectively. Both RF spectra were recorded with 100-kHz resolution, and the spectrum with a 10-MHz span shows an isolated line. The signal-to-noise ratios of each signal at 1560 and 1335 nm are 23 and 10 dB, respectively. The linewidth of the free-running beat note is the convolution of the linewidth of the two CW lasers and depends on their stability. Both linewidths are around 100 kHz so that the phase coherence at 1335 nm is comparable with that at 1560 nm. The RF spectrum of the beat signals between the on-chip OFC and the tunable laser at 1335 nm could not be measured with the SWG without the graphene because the intensity of the SC light component at 1335 nm is about one order magnitude lower. These results show that the spectrum near 1335 nm newly generated from G-SWG becomes an OFC in the frequency domain.

## Conclusion

We have experimentally and numerically demonstrated the enhancement of broadband on-chip OFC generation by decorating an SWG with graphene. The results show that, by precisely controlling length *L_Gr_* and position *P* of the graphene on the SWG, the graphene increases the effective 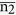 and compresses the laser pulse width. It was clearly shown that the most suitable graphene length L_Gr_ is 200 μm. When it is more than 200 μm, the on-chip OFC bandwidth becomes smaller by TPA. On the other hand, the laser pulse widths before Area 2 for *P* from 100 to 300 μm differ little, while the difference in phases is large. The phase difference influences the laser pulse width in Area 2. The simulation results are reproduced quite well by the experimental results. Although the on-chip OFC becomes wider for *P* of 600 μm, the laser pulse splits. From the experimental and simulation results, it is found that the widest and highest on-chip OFC spectrum without splitting of the laser pulse can be generated for *P* of 300 μm from the SWG incident side and L_Gr_ of 200 μm. Our work indicates the feasibility of a new compact and broadband light source using graphene and silicon-photonics technology. Our method has versatility because the silicon and graphene can be replaced with transparent materials (SiO_2_, Si_3_N_4_, InP, GaAs, etc.) and two-dimensional materials (MoS_2_, MoSe_2_, WS_2_, WSe_2_), respectively. By using these attractive materials, the required pump laser power can be further decreased in integrated photonic circuits. Although SWGs show the influence of TPA with a pump pulse light source at the wavelength of 1.5-μm, Si_3_N_4_ waveguides^32^,^33^ can be used to further increase the output power considerably. Moreover, our method will contribute to applications to future integrated devices because it can control the nonlinear coefficient at an arbitrary place of a waveguide.

## Methods

### Simulations

The propagation of the temporal envelope *A (z, t*) of the electric field of short pulses along the length z of a nonlinear medium is described by the generalized nonlinear Schrödinger equation^29^:


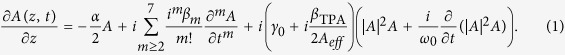


where *α* is the linear propagation loss, *β*_*m*_ is the m-th order dispersion coefficient, *β*_*TPA*_ is the TPA coefficient, *γ*_*0*_* = ω*_*0*_*n*_*2*_/*cA*_*eff*_ is the nonlinear Kerr coefficient, *ω*_*0*_ is the optical frequency, *n*_*2*_ is the nonlinear index coefficient, and *A*_*eff*_ is the effective area. The effective 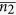 of the G-SWG is calculated for an inhomogeneous cross-section weighted with respect to field distribution^34^,^35^. Note that the most exciting property of graphene is its extremely high third-order nonlinearity (*n*_*2,Gr*_ of 10^−11^~10^−17^ m^2^/W^18^,^20^,^21^,^22^,^36^). There are large differences in the local Kerr coefficient *n*_*2*_ in graphene between theory^37^ and experiment^36^,^38^. Here, the experimentally extracted local Kerr coefficient *n*_*2*___Gr_ of 1 × 10^−13^ m^2^/W and two photon absorption coefficient *β*_*2*_Gr_ of 1 × 10^−7^ m/W in graphene^18^ and *n*_2_Si_ of 4.2 × 10^−18^ m^2^/W and *β_2_Si_* of 8 × 10^−12^ m/W in bulk silicon^17^ were applied to our numerical calculation. Indeed, the simulated spectrum broadening almost completely agrees with our experimental result. The mode distribution was obtained from a mode solver (FIMMWAVE, Photon Design Ltd). The resulting effective 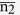
was calculated to be 4.1 × 10^−17^ m^2^/W.

Table 1 shows the values of the G-SWG parameters employed in our study.

## Additional Information

**How to cite this article**: Ishizawa, A. *et al*. Optical nonlinearity enhancement with graphene-decorated silicon waveguides. *Sci. Rep.* **7**, 45520; doi: 10.1038/srep45520 (2017).

**Publisher's note:** Springer Nature remains neutral with regard to jurisdictional claims in published maps and institutional affiliations.

## Supplementary Material

Supplementary Information

## Figures and Tables

**Table 1:** Values of the parameters used for numerical simulation.

Parameter name	Symbol	Value	
SWG length	*L*_*Si*_	2 mm	Experimental value
Graphene length	*L*_*Gr*_	200 μm	Experimental value
Linear loss of G-SWG	*α*_*Gr*_	0.052 dB/μm	Measured
Effective nonlinear refractive index of G-SWG	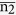	4.1 × 10^−17^ m^2^/W	Calculated
Effective mode area	*A*_eff_	0.144 μm^2^	Calculated
TPA coefficient of G-SWG	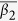	4.5 × 10^−11^ m/W	Calculated
