# Supplementary material for: Optical nonlinearity enhancement with graphene-decorated silicon waveguides
Source: Sci Rep. 2017 Apr 12;7:45520. doi: 10.1038/srep45520 (PMC5388840; doi:10.1038/srep45520)
Supplement: Supplementary Information [file srep45520-s1.doc]

**Supplementary Information**

**Optical nonlinearity enhancement with graphene-decorated silicon waveguides**

Atsushi Ishizawa^1*^, Rai Kou^2,3*^, Takahiro Goto^1,4*^, Tai Tsuchizawa^2,3^, Nobuyuki Matsuda^1,2^, Kenichi Hitachi^1^, Tadashi‍ Nishikawa^4^, Koji Yamada^2,3^, Tetsuomi Sogawa^1^, and Hideki Gotoh^1^

^1^*NTT Basic Research Laboratories, NTT Corporation, 3-1 Morinosato Wakamiya, Atsugi-shi, Kanagawa 243-0198, Japan*

^2^*NTT Nanophotonics Center, NTT Corporation, 3-1 Morinosato Wakamiya, Atsugi-shi, Kanagawa 243-0198, Japan*

^3^*NTT Device Technology Laboratories, NTT Corporation, 3-1 Morinosato Wakamiya, Atsugi-shi, Kanagawa 243-0198, Japan*

^4^*Tokyo Denki University, Department of Electrical and Electronic Engineering, 5 Senjyu Asahi-cho, Adachi-ku, Tokyo 120-8551, Japan*

*^*^These authors contributed equally to this work.*

*e-mail address: ishizawa.atsushi@lab.ntt.co.jp*

The supplementary information contains the following sections:

1. **Device fabrication**
2. **Raman spectroscopy**
3. **Spectral broadening in the graphene-position-controlled G-SWG**

**1. Device fabrication**

A 4-inch silicon-on-insulator (SOI) wafer (220-nm active layer thickness, 3-μm buried oxide layer thickness) was prepared for G-SWG fabrication. The major steps are as follows:

1. SiO_2_ plasma-enhanced chemical vapor deposition (PECVD)
2. Electron-beam lithography for resist patterning
3. Reactive ion etching (RIE) for SiO_2_-hard-mask patterning
4. Electro-cyclotron resonance RIE for SWG patterning
5. [Figure S1(1)] Spin coating poly(methyl methacrylate) (PMMA) onto graphene on Cu substrate (Graphene Platform Ltd., CVD single-layer graphene on Cu foil)
6. [Figure S1(2)] Cu etching in ammonium peroxodisulfate ((NH_4_)_2_S_2_O_8_) solution
7. [Figure S1(3)] Transferring graphene to SWG substrate in deionized water
8. Dissolving PMMA by acetone
9. Spin coating photoresist and photolithography for graphene pattern
10. [Figure S1(4)] Graphene etching by O_2_ plasma RIE
11. Spin coating photoresist and photolithography for SSC pattern
12. SiO_2_ PECVD
13. [Figure S1(5)] Liftoff in acetone


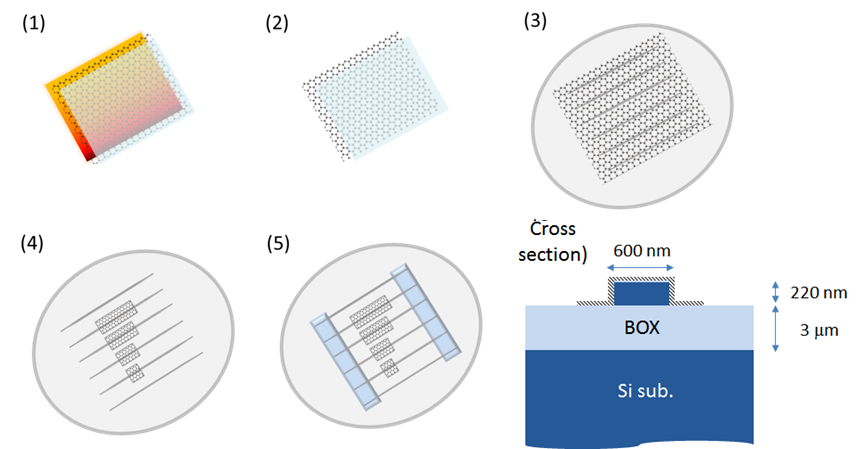


**Fig. S1. Integration process steps for G-SWG and a cross-sectional schematic.**

**2.** **Raman spectroscopy** Transferred graphene quality was evaluated by micro-Raman spectroscopy, which allows us to figure out the major characteristics such as the number of layers, defects in the honeycomb lattice, and impurity doping. A graphene sheet on a silicon waveguide was excited by a 532-nm pumped laser light source to extract the Raman scattering response. The spot diameter was estimated to be around a few micrometers with a ×100 objective lens. A typical spectrum is shown in Fig. S2, where two obvious peaks are observed at G and 2D bands. These wave numbers, 1583 and 2674 cm^-1^, reveal that single-layer and p-doping graphene are dominant components^1, 2^. Moreover, the flat response at the D band (~1350 cm^-1^) corresponds to damage-free ideal graphene.


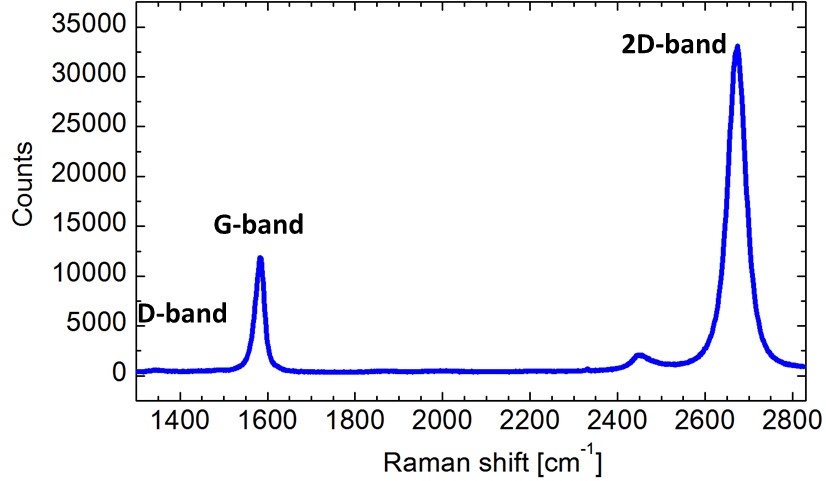


**Fig. S2. Raman spectrum of the graphene on the SWG**

1. **Spectral broadening in the graphene-position-controlled G-SWG**

We simulated the dependence of the OFC intensity on the graphene position in detail by using the generalized nonlinear Schrödinger equation with the split-step Fourier method. As shown in Fig. S3a, we divide the G-SWG into three areas. The graphene is transferred only onto Area 2 of the SWG. Figure S3 (b)-(e) show the laser pulse width and on-chip OFC spectra in each area of the G-SWG for graphene position *P* of 100, 200, 300, and 600 μm. Figure S3 (f) shows the laser phase before Area 2. The laser pulse widths before Area 2 for *P* from 100 to 300 μm differ little, while the difference in phases is large. The phase difference influences the laser pulse width in Area 2. The simulation results are quite well reproduced by the experimental results. Although the on-chip OFC becomes wider for *P* of 600 μm, the laser pulse splits. From experimental and simulation results, it was found that the widest and highest on-chip OFC spectrum without splitting of the laser pulse can be generated for *P* of 300 μm because the laser pulse width becomes smallest in the G-SWGs and strong SPM can occur.


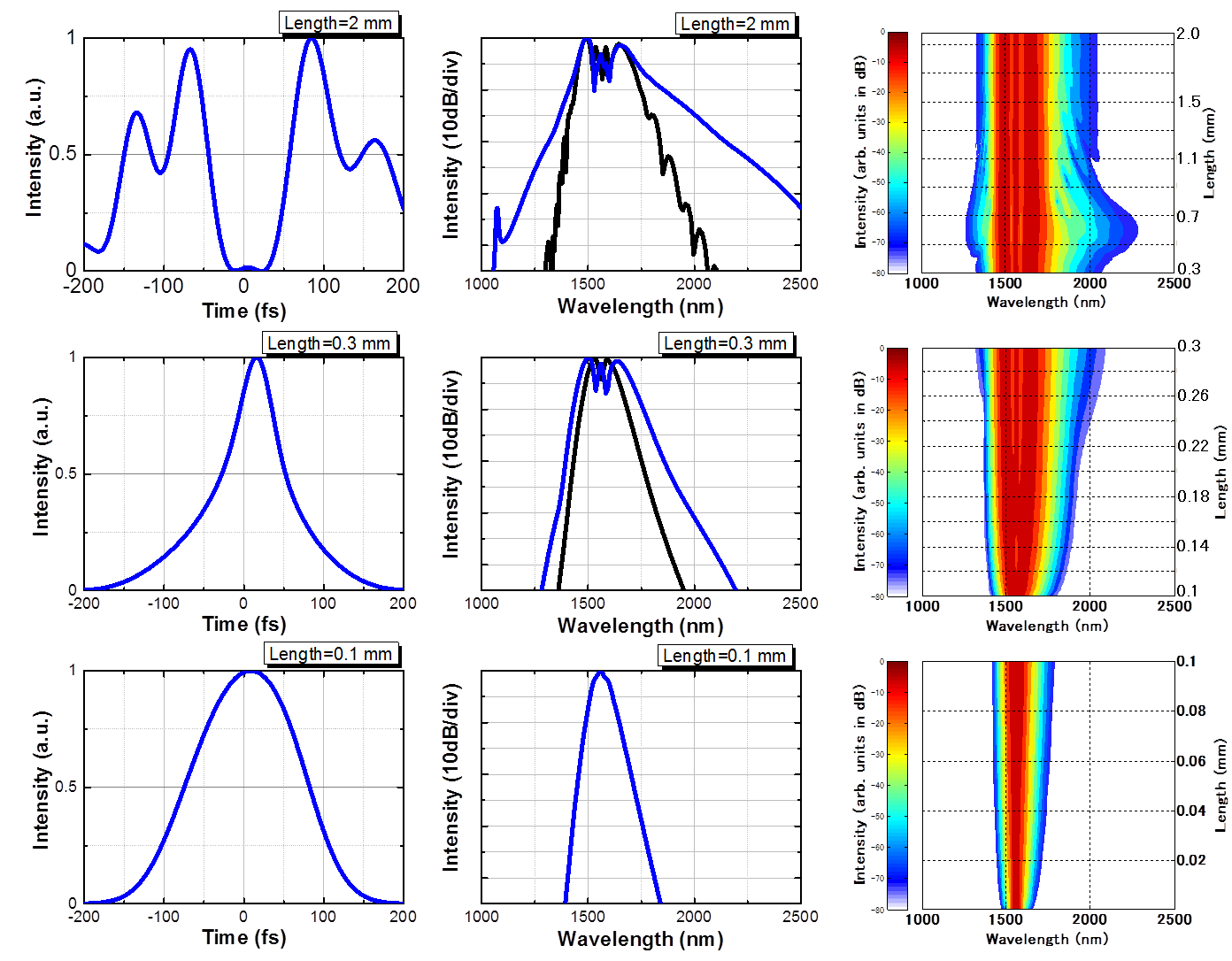

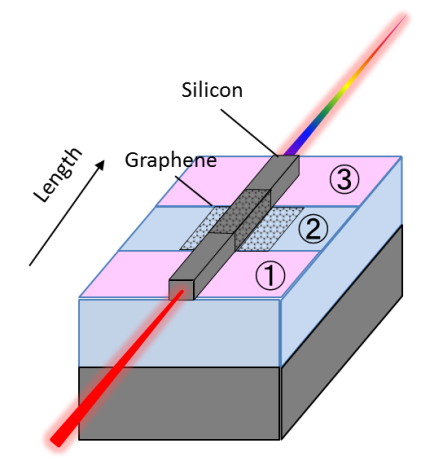


(a)

(b)

**
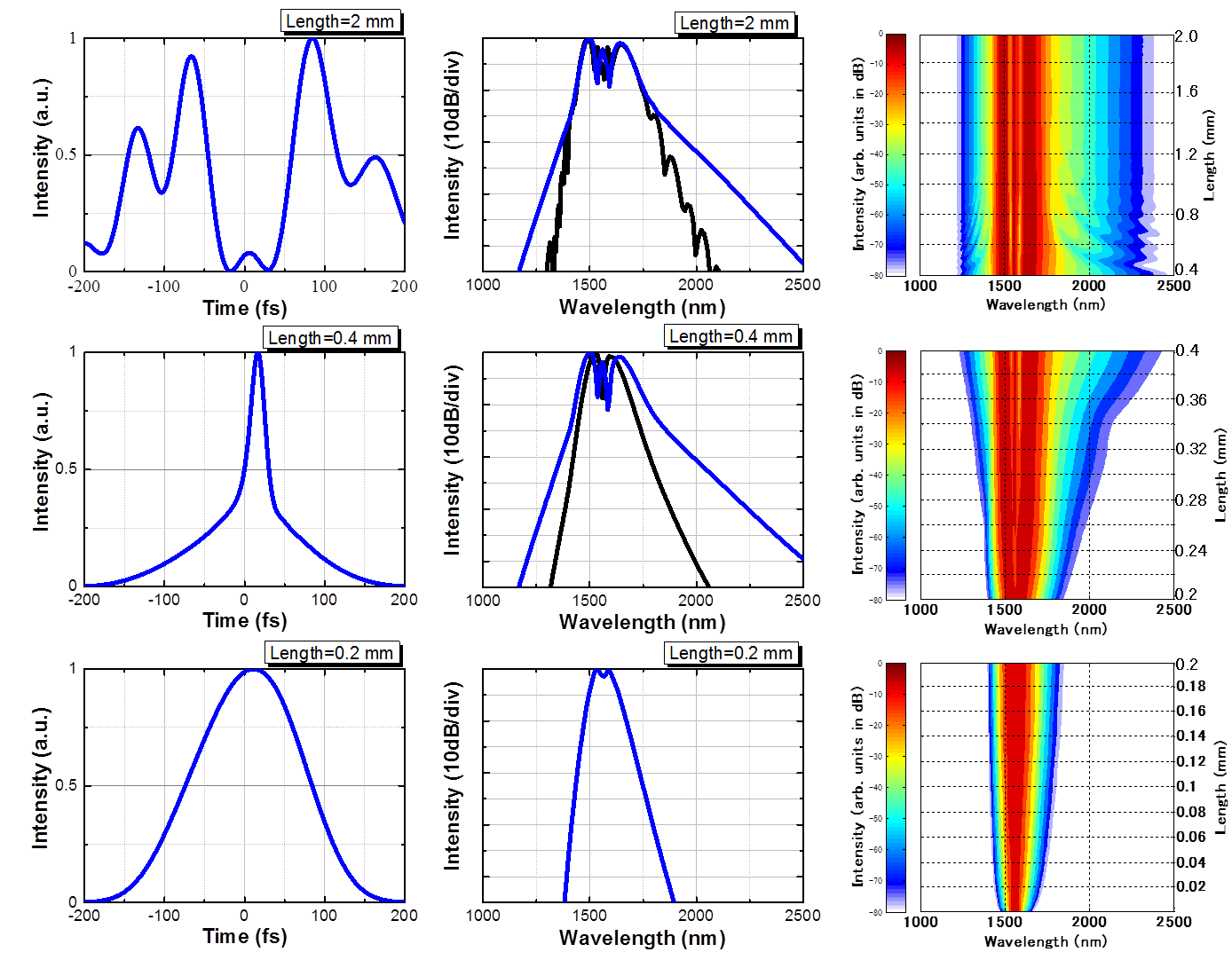
**

(c)

(d)

**
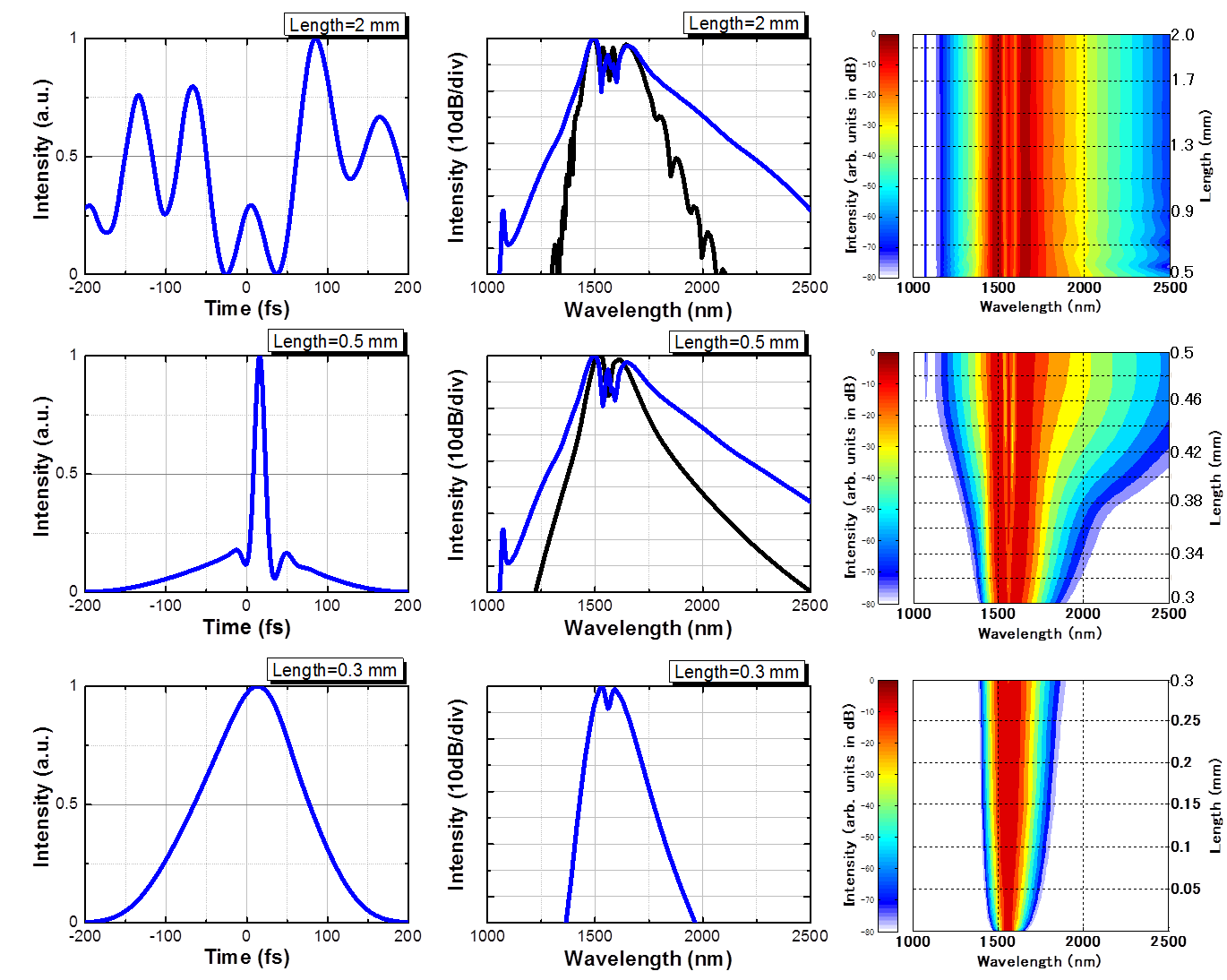
**


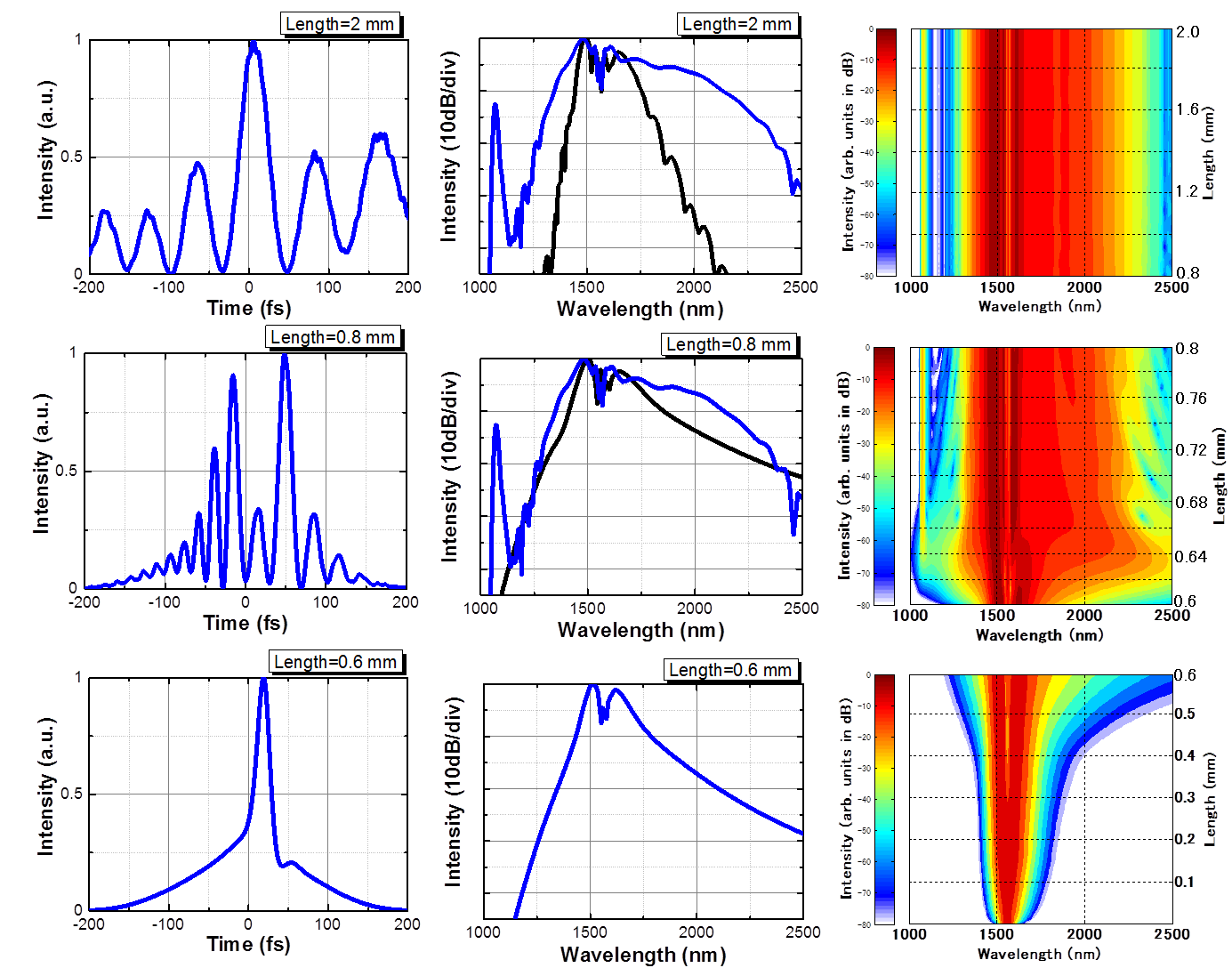


(e)


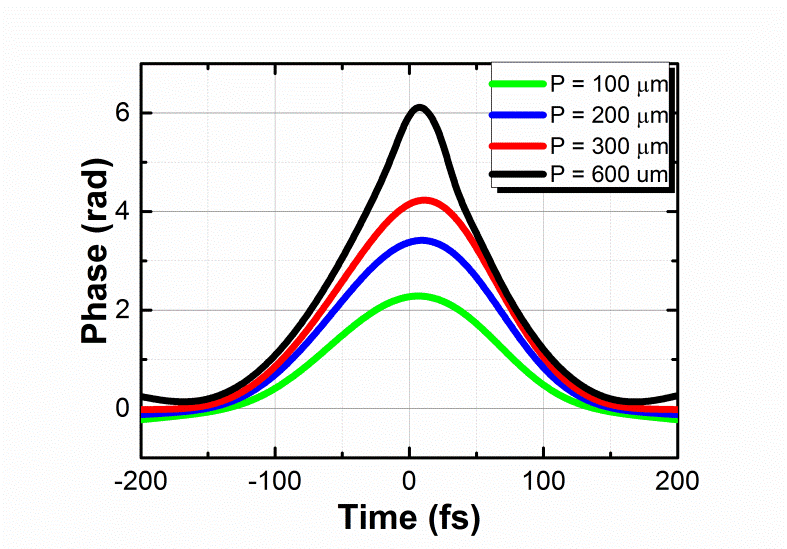


(f)

**Fig. S3. Laser pulse propagation into our fabricated G-SWGs** (**a**) G-SWG divided into three areas, with graphene transferred only onto Area 2 of the SWG. The laser pulse shape in the time domain (left), optical spectrum with (blue) and without (black) (center), and the spectrum evolution (right) in each area are shown for graphene position *P* of (**b**) 100, (**c**) 200, (**d**) 300, and (**e**) 600 μm. (**f**) Laser phases before Area 2 for *P* from 100 to 600 μm.

**References**

1. Dresselhaus, M. S., Jorio, A., Hofmann, M., Dresselhaus, G. & Saito, R. Perspectives on Carbon Nanotubes and Graphene Raman Spectroscopy. *Nano Lett.* **10,** 751-758 (2010).

2. Das, A. *et al.* Monitoring dopants by Raman scattering in an electrochemically top-gated graphene transistor. *Nat. Nanotechnol.* **3,** 210-215 (2008).
